# Supplementary material for: Integrative structure determination reveals functional global flexibility for an ultra-multimodular arabinanase
Source: Commun Biol. 2022 May 16;5:465. doi: 10.1038/s42003-022-03054-z (PMC9110388; doi:10.1038/s42003-022-03054-z)
Supplement: Supplementary file 2 — Supplementary Information [file 42003_2022_3054_MOESM2_ESM.pdf]

Supplementary Information

for

Integrative Structure Determination Reveals Functional Global Flexibility for an Ultra-Multimodular Arabinanase

Shifra Lansky[1*], Rachel Salama[2], Xevi Biarnés[3], Omer Shwartstein[1], Dina Schneidman-Duhovny[4], Antoni Planas[3], Yuval Shoham[2*] and Gil Shoham[1*]

[1]Institute of Chemistry, the Hebrew University of Jerusalem, Jerusalem 91904, Israel

[2]Department of Biotechnology and Food Engineering, Technion, Haifa 3200, Israel

[3]Laboratory of Biochemistry, Institut Químic de Sarrià, Universitat Ramon Llull, Barcelona 08017, Spain

[4]School of Computer Science and Engineering, the Hebrew University of Jerusalem, Jerusalem 91904, Israel

Supplementary Figure 1

(a)

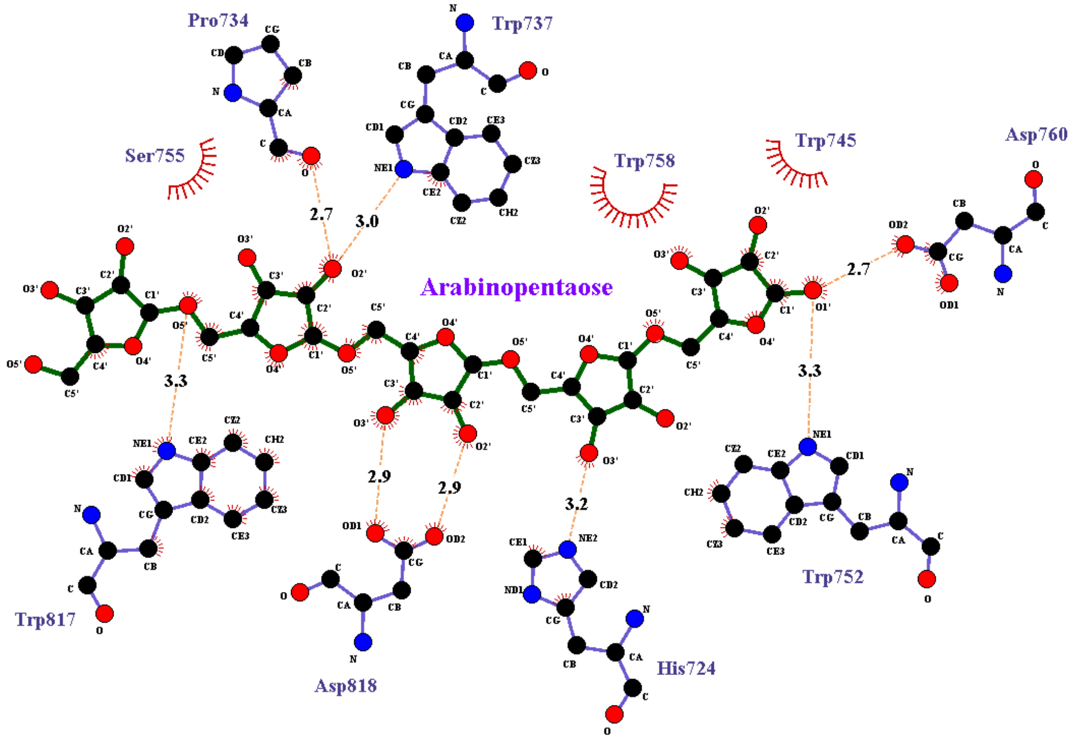

(b)

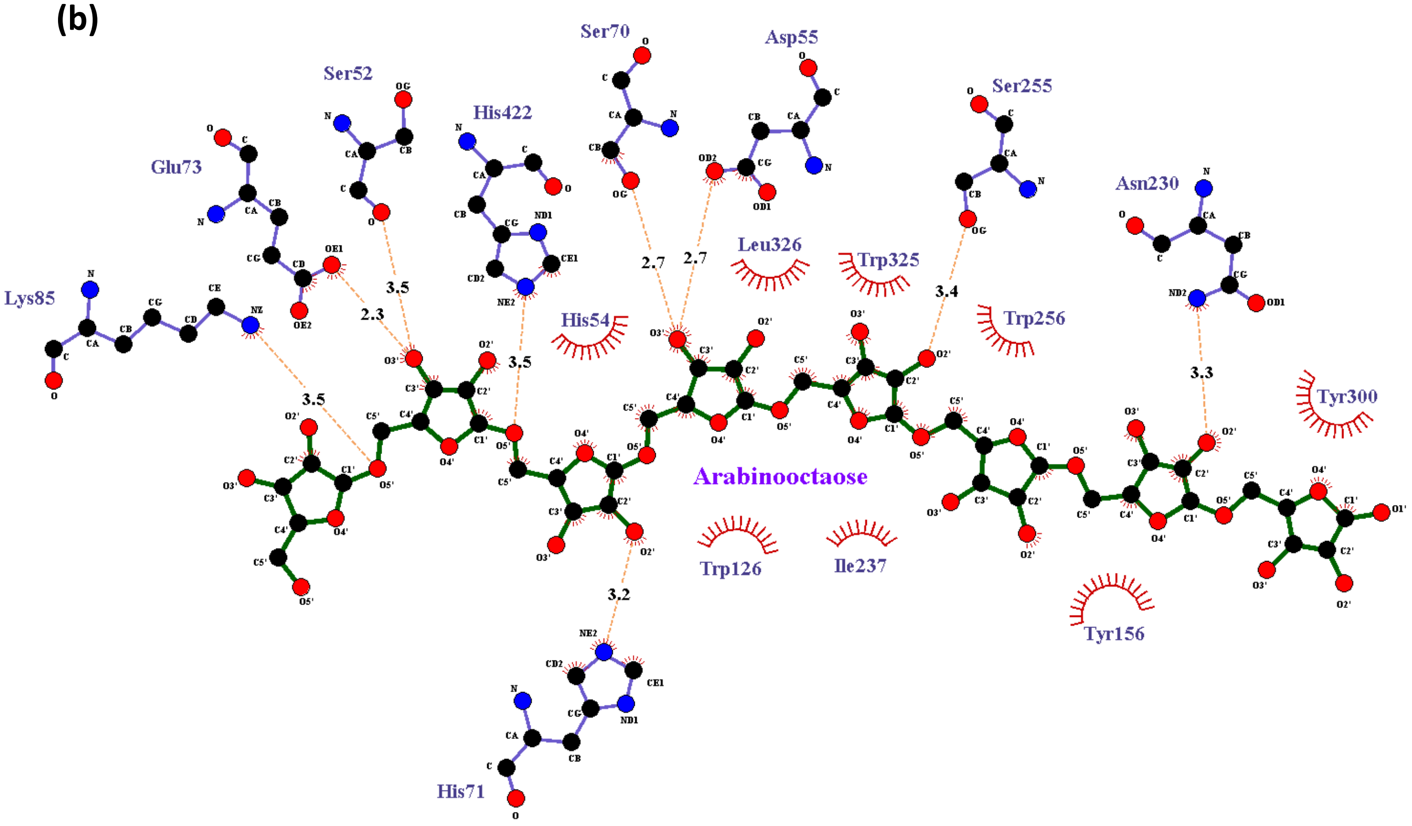

Supplementary Figure 1: The molecular interactions that **(a)** the arabinopentaose molecule forms with the amino acids of Domain4 in the AbnA-Conf1-A5 structure, **(b)** the A8 molecule forms with the active site in the structure of AbnA-D123-A8. Figures were produced with Ligplot++.

Supplementary Figure 2

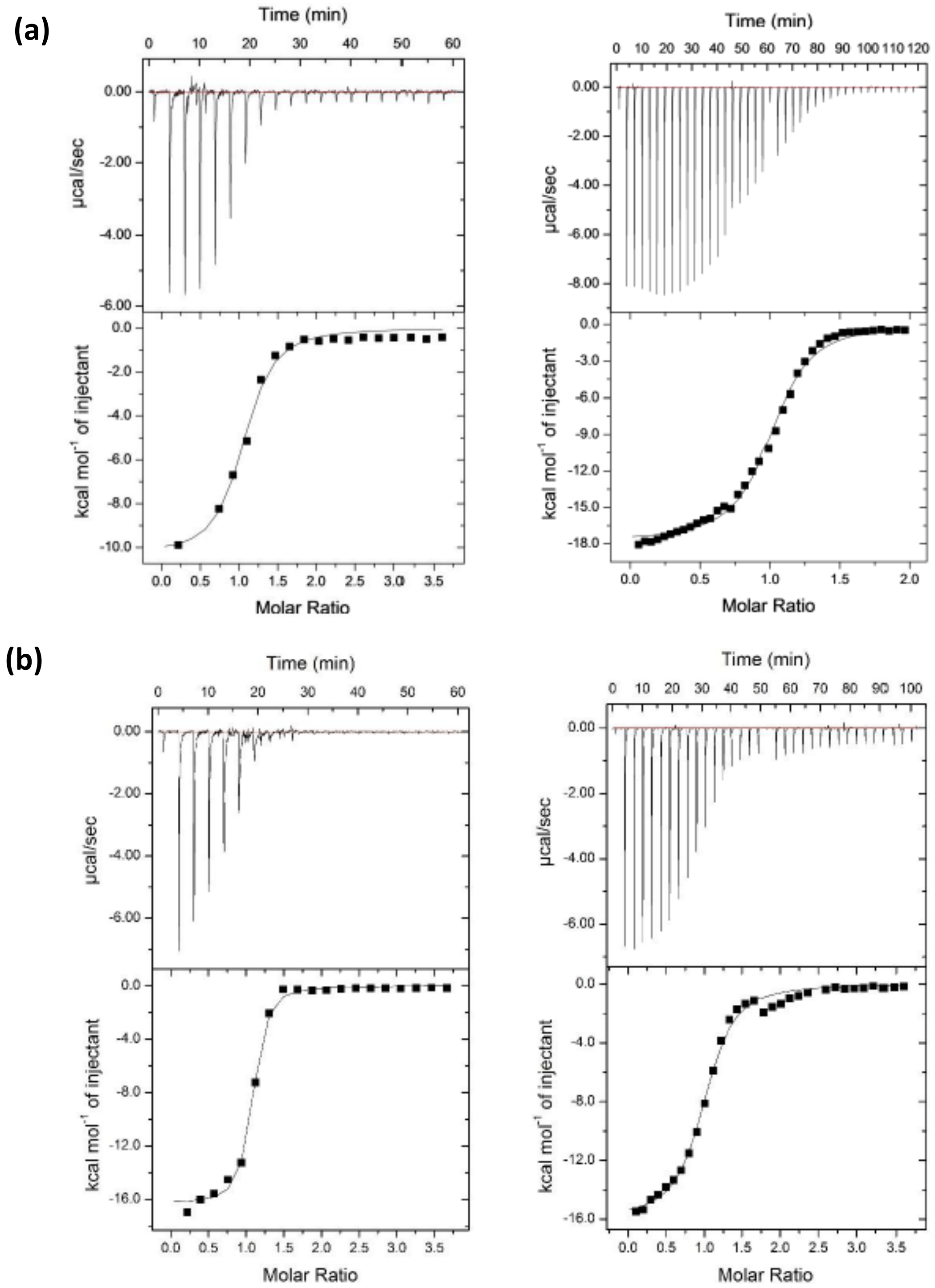

Supplementary Figure 2: ITC curves for the binding of substrates to the different domains of AbnA. **(a)** Domains123 (left) and Domain4 (right) with sugar-beet arabinan. **(b)** Domains123 (left) and Domain4 (right) with linear arabinan. All experiments were performed at 30°C. The top half of each experiment shows the raw data for calorimetric titration of the protein with the ligand, and the lower half displays the integrated injection heats from the upper panel. The solid line is the curve of best fit to a single binding site model that was used to derive the binding parameters.

Supplementary Figure 3

(a)

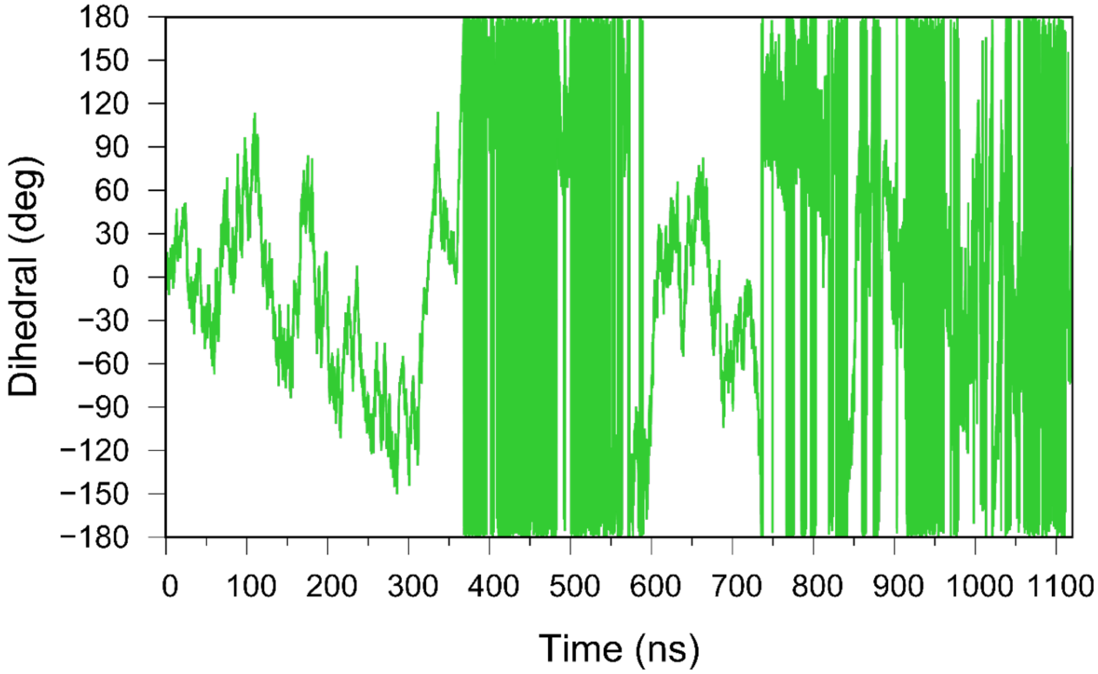

(b)

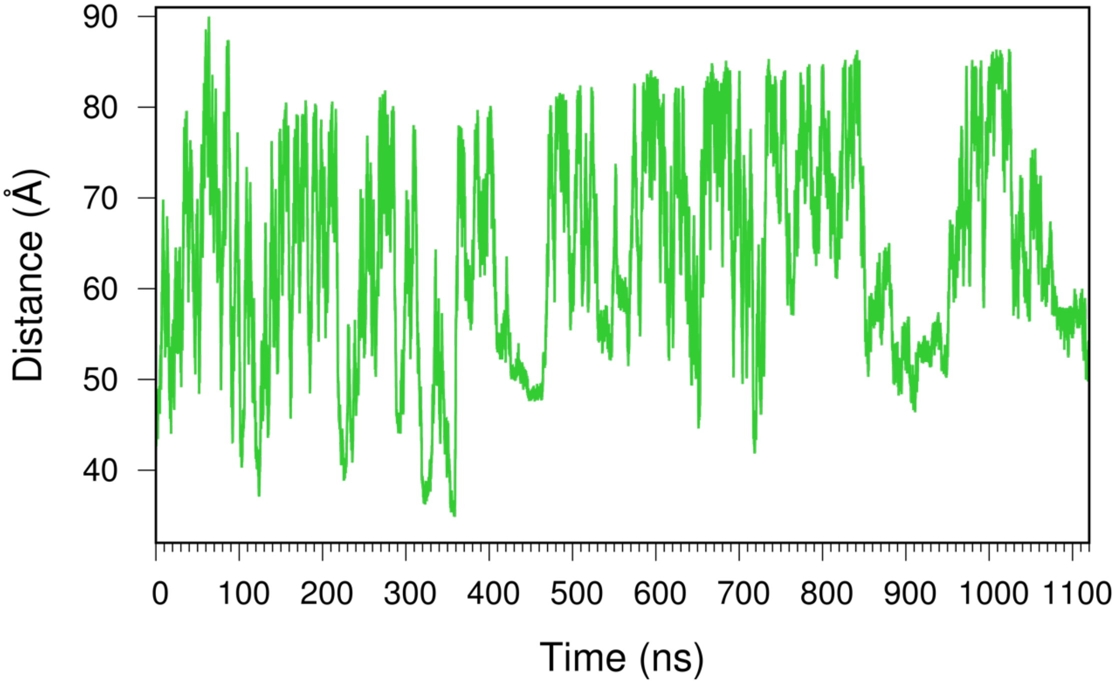

Supplementary Figure 3: Convergence of the metadynamics simulation after 1.12 µs. (a) The distance between Domain 1 and 4 as a function of time. **(b)** The dihedral angle between Domains 1, 2, 3 and 4 as a function of time.

Supplementary Figure 4

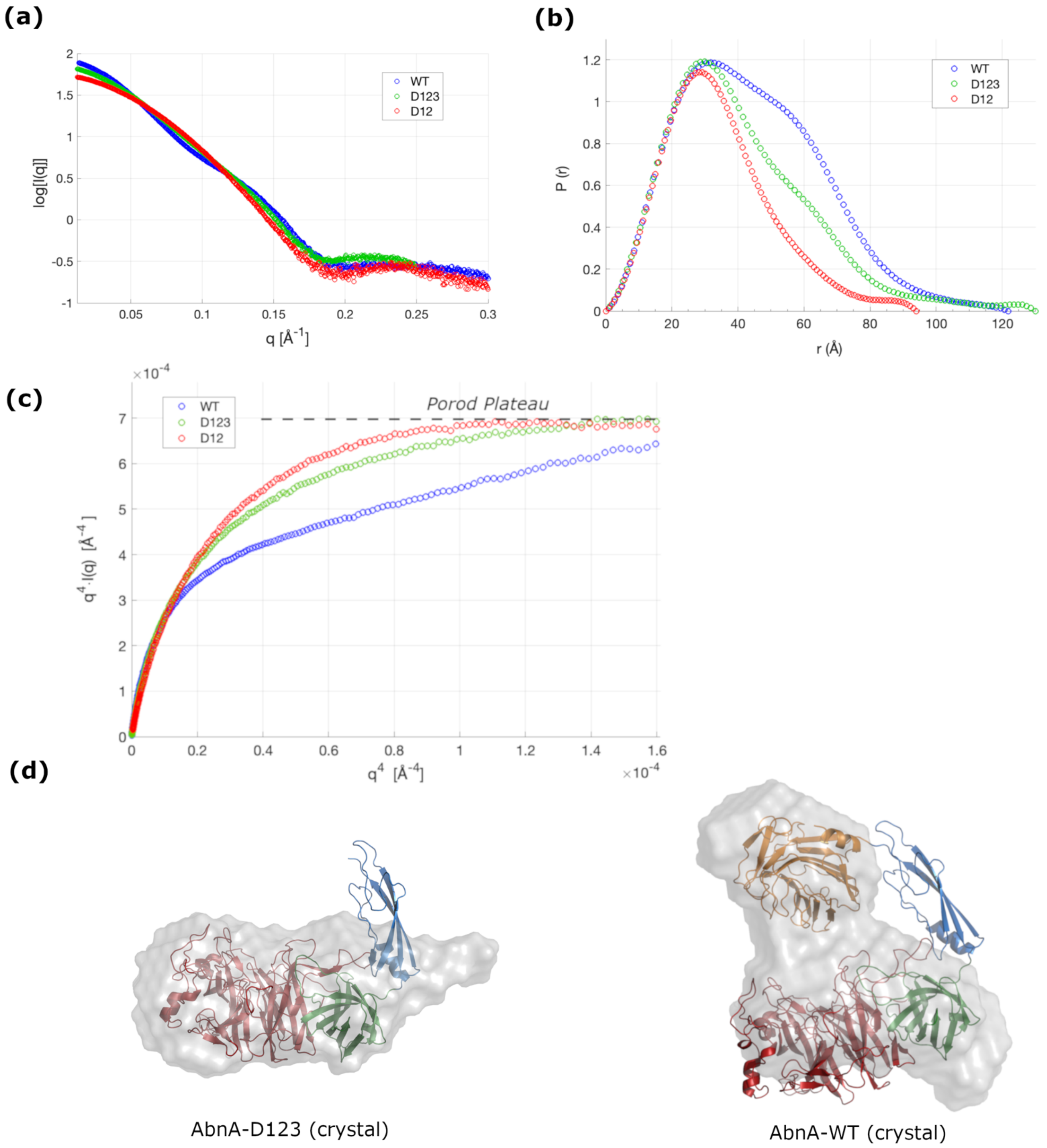

Supplementary Figure 4: SAXS measurements for AbnA-WT, AbnA-D123, AbnA-D12. Comparison between the scattering curves **(a)**, pair-distribution functions (P(r) **(b)**, and Porod-Debye plots (**c**) of AbnA-WT (blue), AbnA-D123 (green), and AbnA-D12 (red). **(d)** Superposition of the AbnA-D123 and AbnA-WT (AbnA-Conf1) crystal structures onto the averaged SAXS envelopes obtained from their data.

Supplementary Figure 5

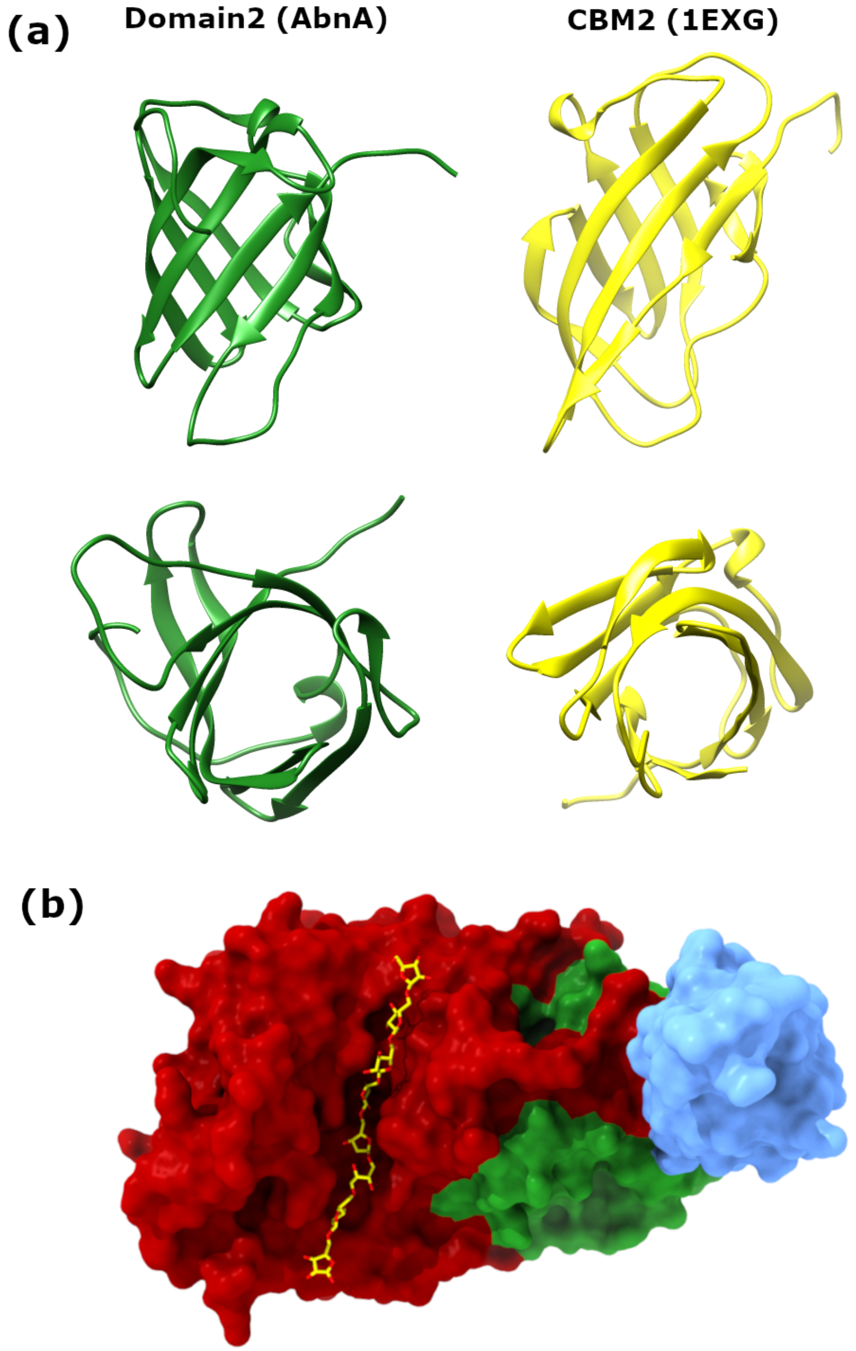

Supplementary Figure 5: a. Qualitative comparison between the fold of Domain2 in the AbnA structure to the crystal structure of a CBM2 family protein (pdb 1EXG), demonstrating overall structural similarities. **b.** Surface representation of the AbnA-D123-A8 structure (Domain 1- red; Domain2- green; Domain3- blue).
